# Novel Antimicrobial Cellulose Fleece Inhibits Growth of Human-Derived Biofilm-Forming Staphylococci During the SIRIUS19 Simulated Space Mission

**DOI:** 10.3389/fmicb.2020.01626

**Published:** 2020-07-29

**Authors:** Daniela Wischer, Dominik Schneider, Anja Poehlein, Friederike Herrmann, Harun Oruc, Junias Meinhardt, Olaf Wagner, Rameez Ahmed, Sergey Kharin, Natalia Novikova, Rainer Haag, Rolf Daniel, Elisabeth Grohmann

**Affiliations:** ^1^Faculty of Life Sciences and Technology, Department of Microbiology, Beuth University of Applied Sciences, Berlin, Germany; ^2^Department of Genomic and Applied Microbiology, Göttingen Genomics Laboratory, Institute of Microbiology and Genetics, Georg-August-University Göttingen, Göttingen, Germany; ^3^Institute of Chemistry and Biochemistry, Freie Universität Berlin, Berlin, Germany; ^4^Institute of Biomedical Problems (IBMP), Moscow, Russia

**Keywords:** antimicrobial material, SIRIUS, isolated environment, space microbiology, antibiotic resistance, human-commensal bacteria

## Abstract

Two novel antimicrobial surface coatings were assessed for their lasting antibacterial effect under simulated space conditions during the SIRIUS-19 study. Because long-term space travel can affect the human immune system, astronauts are particularly susceptible to infectious disease. Moreover, the space flight environment can alter the composition of microbial communities within the spacecraft and increase bacterial virulence and resistance to antibiotics. In addition to protecting the crew from infection by human pathogens, prevention and elimination of bacterial contamination is important to avoid corrosion and damage of the technical equipment. The antimicrobial coating AGXX^®^ consists of micro-galvanic cells composed of silver and ruthenium which damage bacterial cells through the release of reactive oxygen species. Over the last years, several studies on the antimicrobial effect of AGXX^®^ have demonstrated an effective inhibition of growth and even complete elimination of many pathogenic bacteria – including multiresistant microorganisms – as well as their biofilms. The second antimicrobial coating, GOX, consists of chemically modified graphene oxide. Through a positive surface charge and its flexible scaffold, GOX can multivalently bind and immobilize bacteria via electrostatic attraction. Here, AGXX^®^ and GOX were applied to non-metallic carriers not previously tested. The antimicrobial coated materials, as well as uncoated control samples, were exposed in the SIRIUS artificial space module and analyzed at different time points during the 4-months isolation study. Survival and growth of airborne heterotrophic, aerobic bacteria on the surfaces were assessed by cultivation-based methods, employing growth conditions suitable for potential human pathogens. Human-associated, biofilm-forming *Staphylococcus* spp. (*S. hominis*, *S. haemolyticus*, and *S. epidermidis*) strongly dominated at all time points, most were resistant against erythromycin, kanamycin, and ampicillin. AGXX^®^ coatings completely inhibited growth of these opportunistic pathogens on all tested surface materials. Particularly, AGXX^®^-cellulose fleece achieved a clear reduction in bacterial load able to recover post contact. GOX-cellulose fleece effectively immobilized bacteria. Sequence analysis of 16S rRNA gene amplicons revealed that the isolated *Staphylococcus* spp. did not dominate the overall bacterial community, accounting for only 0.1–0.4% of all sequences. Instead, molecular data revealed *Lactobacillus*, *Comamonas*, *Pseudomonas*, *Sporosarcina*, and *Bacillus* as the dominant genera across all samples and time points.

## Introduction

Space stations are confined, closed habitats with conditions that pose unique challenges to the human body and can impact the health and performance of astronauts. These stressors include environmental factors such as microgravity and radiation, as well as social stresses including isolation and sleep deprivation. Among other health effects, space flight can significantly alter immune response, and cause reactivation of latent viruses (Stowe et al., 2001; Pierson et al., 2005; Sonnenfeld, 2005; Taylor, 2015; Crucian et al., 2018; Wu et al., 2018).

Importantly, space flight conditions affect not only humans but also the microorganisms within spacecrafts, including the astronauts’ microbiome (Jiang et al., 2019; Voorhies et al., 2019). Bacteria exposed to space flight conditions have been shown to respond with changes in cell morphology and cell wall thickness (Novikova et al., 2006; Zea et al., 2017), an increase in secondary metabolite and extracellular polysaccharide production (Mauclaire and Egli, 2010), increased growth rate, biofilm formation, enhanced virulence, and increased resistance to antibiotics (Rosenzweig et al., 2010; Kim et al., 2013; Taylor, 2015; Aunins et al., 2018; Fajardo-Cavazos et al., 2018; Urbaniak et al., 2018; Bai et al., 2019; Liu et al., 2019; Mora et al., 2019; Zhang et al., 2019).

Without access to medical experts, any bacterial infections arising during space missions require treatment with broad-spectrum antibiotics which further facilitates the development of antibiotic resistance (Barratt and Pool, 2008). Therefore, preventing bacterial infections of crew members during space missions is paramount. In addition to health concerns, bacterial colonization of surfaces within the spacecraft can also lead to corrosion and damage of technical equipment (Klintworth et al., 1999; Alekhova et al., 2005).

Bacterial resistance to antibiotics is becoming a serious global threat, necessitating research into effective antimicrobial alternatives. Due to its high bactericidal effect, especially silver (both in ionic and nanoparticulate form) is frequently used in wound dressings, medical devices, water treatment, textile fibers, food packaging, and anti-fouling paints (Vonberg et al., 2008; Greulich et al., 2012; Sim et al., 2018; Vila Domínguez et al., 2020). However, the extensive use of silver nanoparticles has raised concerns over the health risks and environment impacts of these products (Chopra, 2007; Greulich et al., 2012; Ostaszewska et al., 2018; Liao et al., 2019). Additionally, resistance to silver compounds in *Salmonella* spp., *Escherichia coli*, and *Pseudomonas aeruginosa* is an increasing problem (Gupta et al., 1999; Silver, 2003; Panáček et al., 2018). With this rising resistance against both conventional antibiotics and antimicrobial metals, there is an urgent need for alternative ways to fight bacterial infection.

AGXX^®^ is an antimicrobial surface coating that combines the two noble metals silver (Ag) and ruthenium (Ru) into micro-galvanic cells conditioned with ascorbic acid. The coating can be applied to a wide range of carrier materials, including steel, glass, ceramics, and organic polymers (Guridi et al., 2015; Clauss-Lendzian et al., 2018; Loi et al., 2018; Vaishampayan et al., 2018). The antimicrobial properties of AGXX^®^ mainly result from the release of reactive oxygen species (ROS) such as H_2_O_2_ and OH which are formed through a series of redox reactions (Guridi et al., 2015; Clauss-Lendzian et al., 2018; Loi et al., 2018). AGXX^®^ is active against a wide range of microorganisms, including both Gram-positive and Gram-negative bacteria, filamentous fungi, yeasts, and some viruses (Guridi et al., 2015; Landau et al., 2017; Sobisch et al., 2019). The antibacterial effect of AGXX^®^ is also successful against multiresistant pathogens: AGXX^®^ killed multiresistant clinical strains of *E. faecalis*, *E. faecium*, *S. epidermidis* as well as methicillin-resistant *Staphylococcus aureus* (MRSA) and strongly reduced growth of *Legionella erythra* and the highly pathogenic Shiga toxin-producing *E. coli* O104:H4 strain (Guridi et al., 2015; Mayer et al., 2016; Clauss-Lendzian et al., 2018; Vaishampayan et al., 2018). Studies using next generation RNA sequencing revealed that exposure to AGXX^®^ induces a broad general stress response in *E. faecalis* with up-regulation of heat shock genes and a strong oxidative stress response (Clauss-Lendzian et al., 2018). Similar studies of MRSA strains exposed to AGXX^®^ revealed a thiol-specific oxidative stress response (Loi et al., 2018) and repression of genes for biofilm formation, virulence factors, and quorum sensing systems (Vaishampayan et al., 2018). To date, bacterial resistance against AGXX^®^ has not been observed, making AGXX^®^ a promising broad-spectrum antimicrobial. In a recent study carried out on the International Space Station (ISS), AGXX^®^ was shown to strongly reduce bacterial growth on surfaces prone to contamination, particularly by biofilm-forming Staphylococci (Sobisch et al., 2019).

Building on results by Sobisch et al. (2019) we investigated the long-term antimicrobial effect of AGXX^®^ during SIRIUS19, a 4-months simulated space mission taking place inside the NEK facility (referred to as SIRIUS module in the text), an on-ground artificial space habitat at the Institute for Medical and Biomedical Problems at the Russian Academy of Sciences (IBMP-RAS) in Moscow, Russia. In addition to AGXX^®^, we investigated the effect of a second, recently developed antimicrobial coating based on graphene oxide (GO). The surface charge of the GO sheets was switched to positive by functionalization with polymers carrying positive charged groups. The resulting positively charged flexible micrometer-sized sheets are named GOX. A physical property that almost all bacteria share is their overall negative surface charge (Dickson and Koohmaraie, 1989; Silhavy et al., 2010). Positively charged surfaces can therefore promote adhesion and immobilization of bacterial cells (Gottenbos, 2001). GOX can be applied to a wide range of substrates and has been reported to exhibit antibacterial activity toward both Gram-positive and Gram-negative bacteria (*E. coli* and *S. aureus*) (Ahmed et al., 2020).

We investigated the inhibiting effect of AGXX^®^ and GOX on surface colonization by airborne, potentially pathogenic, bacteria during the SIRIUS19 isolation study. Since these organisms are most commonly derived from the astronauts’ themselves, we exposed the antimicrobial materials in an area experiencing high human traffic and physical activity by the crew members. Applications of the two antimicrobial coatings to three different, non-metallic carrier materials were tested. Bacterial communities surviving on the materials were analyzed after 1, 2, and 4 months through a combination of culture-based analyses (selecting for aerobic, heterotrophic bacteria) and 16S rRNA gene amplicon sequencing. Medically relevant bacterial isolates were characterized with regards to their antibiotic resistance profiles and biofilm formation.

## Materials and Methods

### Preparation and Exposure of Antimicrobial Materials

**FIGURE 1:**
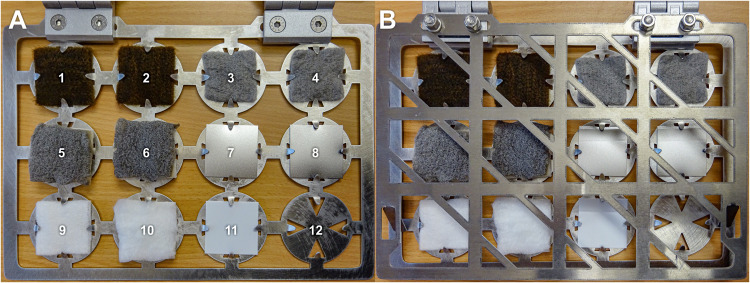
Target book containing antimicrobial coated and uncoated sample materials (size of samples: 4 × 4 cm). **(A)** open; **(B)** closed. 1 and 2, cellulose fleece with AGXX^®^; 3 and 4, cellulose fleece with GOX; 5 and 6, fibrous web fleece with AGXX^®^; 7 and 8: polypropylene with AGXX^®^; 9, uncoated cellulose fleece; 10, uncoated fibrous web fleece; 11, uncoated polypropylene; and 12, empty.

Four different antimicrobial materials were tested: (i) fibrous web fleece (made of polyester fibers) with AGXX^®^, (ii) cellulose fleece with AGXX^®^, (iii) cellulose fleece with GOX, and (iv) polypropylene plate coated with AGXX^®^. Uncoated samples of each carrier material were used as controls. AGXX^®^-coated materials were provided by Largentec GmbH, Berlin, Germany. GOX-coated materials were supplied by the Institute of Chemistry and Biochemistry, Freie Universität Berlin, Germany. A total of 16 cm^2^ of the sample materials were sterilized by autoclaving at 121°C for 20 min (fibrous web fleece, cellulose fleece) or soaking in 70% isopropyl alcohol for 30 min and drying at 60°C (polypropylene plastic) and mounted onto specially designed aluminum frames (target books, Figure 1) in duplicates. Two samples per material were mounted: one for cultivation experiments and one for molecular analysis. Three sets of target books, one for each time point (*t*1 = 1 month, *t*2 = 2 months, and *t*3 = 4 months, thus representing a cumulative bacterial load) were fixed on the walls of the crew’s exercise room (high human traffic and high humidity) inside the SIRIUS facility at the IMBP, Moscow, Russia. In parallel, a reference experiment with the same materials and time points was set up inside a non-isolated control environment with high human traffic (microbiology laboratory at Beuth University of Applied Sciences, Berlin, Germany). At the end of each time point, samples were placed into sterile zip lock bags, transported to Berlin at 4°C and processed immediately after arrival to the laboratory.

### Isolation of Bacteria and Phylogenetic Affiliation

Conditions for bacterial isolation were chosen suitable for aerobic, heterotrophic potential human pathogens, most commonly deriving from the crew’s microbiome. R2A agar was used in order to accommodate also slower-growing bacteria and maximize diversity of isolates. To analyze the growth-inhibiting effect of the antimicrobial materials and to recover bacteria that survived on the surfaces following 1, 2, or 4 months exposure, the sample materials were imprinted onto R2A agar plates, allowing for transfer of cells adhering to the material. Three plates were prepared per sample, the material was pressed onto the agar with the exposed side facing down and removed after a few seconds. An additional plate was prepared for each sample where the material was not removed but remained in direct contact with the agar throughout incubation. All plates were incubated at 37°C for 24–48 h. Morphologically distinct colonies were cultivated at 37°C until pure isolates were obtained. Isolates showing weak growth on R2A were cultured on LB agar (*Staphylococci*) or glucose yeast malt extract (GYM) agar (*Actinomycetes*). Phylogenetic affiliation of isolates was carried out by matrix-assisted laser desorption ionization time-of-flight mass spectrometry (MALDI-TOF MS, Bruker Daltonics MALDI Biotyper system, Bremen, Germany) following extraction of proteins with ethanol and formic acid according to the manufacturer’s instructions (Bruker MALDI Biotyper 3.1 User Manual Revision 1, D2.2). Mass spectra were compared with the MALDI-BDAL Database (Version 3.1, 7,311 entries). According to the manufacturer, the following identification scores were used: 2.0–2.299 = secure genus identification, probable species identification; and 2.3–3.0 = highly probable species identification. Scores ≥ 2.0 were considered as acceptable species-level identification (Schulthess et al., 2013). Each isolate was run at least three times. If identification with MALDI-TOF MS failed, isolates were phylogenetically affiliated by 16S rRNA gene sequencing (Eurofins Genomics Germany GmbH). Colony PCR was conducted as described in the section “Bacterial Colony PCR Assays.” Phylogenetic affiliation of the 16S rRNA gene sequences was performed with the BLAST sequence analysis tool (Altschul et al., 1990). 16S rRNA gene sequences were compared against the nt database for initial classification and subsequently aligned against validated type strains of relevant species as specified in the List of Prokaryotic names with Standing in Nomenclature (LPSN) online resource^1^ (Parte, 2018). The isolates are denominated according to following scheme: (i) the carrier material and (ii) the antimicrobial coating they were isolated from, (iii) the study site, (iv) the exposure time (1, 2, or 3 for time points t1, t2, or t3), and (v) the order of isolation.

### Biofilm Screening Assay

Biofilm formation test was carried out according to Vaishampayan et al. (2018). Briefly, bacterial isolates grown overnight in LB medium at 37°C with shaking (150 rpm) were diluted to an initial OD_600_ of 0.05 and re-grown to exponential phase (OD_600_ ∼1.5). Cultures were transferred to translucent 96-well microtitration plates (Carl Roth^®^ GmbH & Co. KG) in replicates of eight and incubated at 37°C for 24 h. After careful removal of planktonic cultures, the wells were washed twice with 12 mM phosphate buffered saline (pH 7.4) and dried at 55°C for 1 h. Biofilms were stained with 200 μL 0.4% crystal violet solution and briefly rinsed with demineralized water. Biofilm formation was measured at 570 nm (OD_570_) in a SpectraMax^®^ 384 Plus Microplate Reader (Molecular Devices). *Staphylococcus aureus* 04-02981, a strong biofilm former, was used as a positive control, LB medium was used as negative control. Means of five values each and three biological replicates were used. The following criteria were used for the interpretation of the results, ODc = negative control; OD ≤ ODc = non-adherent, ODc ≤ OD ≤ (2 × ODc) = weakly adherent, (2 × ODc) < OD ≤ (4 × ODc) = moderately adherent, (4 × ODc) < OD = strongly adherent (Nyenje et al., 2013).

### Antibiotic Disk Diffusion Method

Antibiotic resistance of the isolates was analyzed with the disk diffusion method (disks from Oxoid^TM^) on Mueller Hinton agar according to guidelines of the European Committee on Antimicrobial Susceptibility Testing^2^ (EUCAST, version 10.0). Sixteen medically relevant antibiotics were selected: ampicillin, oxacillin, ciprofloxacin, ofloxacin, norfloxacin, nalidixic acid, imipenem, meropenem, gentamicin, kanamycin, tetracycline, tigecycline, clindamycin, erythromycin, vancomycin, and nitrofurantoin. Of those, isolates were only screened against the ones recommended for the genus by EUCAST (Supplementary Table 2). Since no criteria for sensitivity testing were available for *Roseomonas* or *Paracoccus*, EUCAST criteria for *Pseudomonas* spp. were used. Each test was performed in triplicates. Multidrug-resistant (MDR) strains were defined as non-susceptible to at least one antimicrobial agent in three or more antimicrobial categories as proposed by Magiorakos et al. (2012).

### DNA Extraction

Metagenomic DNA was extracted from coated and uncoated cellulose fleece samples using the NucleoSpin^®^ Soil DNA extraction kit (Macherey-Nagel). For removal of cells from the material, the cellulose squares were cut into small pieces, suspended in phosphate buffered saline (pH 7.4), thoroughly vortexed for 5 min and incubated in an ultrasound bath (Sonorex RK 106S, Bandelin) for 20 min. Cellulose fibers were removed by passing the suspension through sterile syringes. The filtrate was pelleted by centrifugation at 18,500 × *g* for 30 min in a Thermo Scientific^TM^ Heraeus^TM^ Multifuge^TM^ X3 (Thermo Scientific^TM^). Cell pellets were resuspended with lysis buffer SL2 from the extraction kit, transferred to NucleoSpin^®^ Soil bead tubes and processed according to the manual.

### Bacterial Colony PCR Assays

Details of all primer sets can be found in Supplementary Table 1. For 16S rRNA gene colony PCR assays the primer set 27_fwd/536_rev was used. A single bacterial colony was suspended in 200 μL sterile, nuclease-free water and incubated at 95°C for 5 min. A total of 1 μL was used as template for a 10 μL PCR reaction. PCR reactions contained 0.25 U Taq-Polymerase (VWR), 1 μL 10× PCR reaction buffer S (VWR), 0.2 μM of each primer, 250 μM of each deoxynucleoside triphosphate (dNTP), 0.6 μg/μL bovine serum albumin (BSA), and 1 μL template DNA. All DNA amplifications were carried out in a S10000^TM^ Thermal Cycler (BIO-RAD). Thermal cycling conditions for colony PCR were as follows: initial denaturation for 1 min at 95°C, 30 cycles at 95°C for 30 s, 30 s at 55°C, and 10 s at 72°C, and a final extension at 72°C for 5 min.

### Amplicon Sequence Analysis

#### PCR Assays

For amplicon sequencing, bacterial 16S rRNA genes were amplified using primer set S-D-Bact-0341-b-S-17-N/S-D-Bact-0785-a-A-21-N (Herlemann et al., 2011; Klindworth et al., 2013; Supplementary Table 1) and the Phusion^®^ High Fidelity PCR kit (New England Biolabs). A total of 50 μL PCR reactions were carried out in triplicates. Each reaction contained 1 U Phusion^®^ high-fidelity DNA-polymerase, 10 μL 5× Phusion GC buffer, 0.2 mM MgCl_2_, 0.2 μM of each primer, 200 μM of each dNTP, 0.6 μg/μL BSA, 5% dimethylsulfoxide (DMSO), and 1–3 ng template DNA. Thermal cycling scheme for bacterial amplicons was as follows: initial denaturation for 1 min at 98°C, 30 cycles at 98°C for 45 s, 45 s at 57°C, and 30 s at 72°C, and a final extension at 72°C for 5 min. The resulting PCR products were analyzed and quantified by agarose gel electrophoresis, purified using the NucleoMag^®^ 96 PCR clean-up kit (Macherey-Nagel) and pooled for amplicon sequencing.

#### Library Preparation and Sequencing

PCR products were used to attach indices and Illumina sequencing adapters using the Nextera XT Index kit (Illumina, San Diego). Index PCR was performed using 5 μL of template PCR product, 2.5 μL of each index primer, 12.5 μL of 2× KAPA HiFi HotStart ReadyMix and 2.5 μL PCR grade water. Thermal cycling scheme was as follows: 95°C for 3 min, eight cycles of 30 s at 95°C, 30 s at 55°C, and 30 s at 72°C and a final extension at 72°C for 5 min. Quantification of the products was performed using the Quant-iT dsDNA HS assay kit and a Qubit fluorometer (Invitrogen GmbH, Karlsruhe, Germany) following the manufacturer’s instructions. MagSi-NGS^PREP^ Plus Magnetic beads (Steinbrenner Laborsysteme GmbH, Wiesenbach, Germany) were used for purification of the indexed products as recommended by the manufacturer and normalization of all libraries to the same concentration was performed using the Janus Automated Workstation from Perkin Elmer (Perkin Elmer, Waltham, MA, United States). Sequencing was conducted using Illumina MiSeq platform using dual indexing and MiSeq reagent kit v3 (600 cycles) as recommended by the manufacturer.

#### Sequence Processing and Analyses

Demultiplexing and clipping of adapter sequences from the raw amplicon sequences were performed with the CASAVA software (Illumina). The program fastp (v0.20.0) (Chen et al., 2018) was used for quality-filtering and included removal of sequences with a minimum phred score of 20, a minimum length of 50 base pairs, soft clipping of low quality base pairs with a sliding window size of four bases and phred score of 20, read correction by overlap and adapter removal of the Illumina Nextera primers. Quality-filtered paired-end reads were merged with the paired-end read merger (PEAR, v.0.9.11) (Zhang et al., 2014) with default settings. Additionally, forward and reverse primer sequences were removed with cutadapt (v2.5) (Martin, 2011) with default settings. Sequences were then ordered by length (sequences ≤ 300 bp were removed) and dereplicated by vsearch (version 2.14.1; Rognes et al., 2016). Denoising was performed with the UNOISE3 module of vsearch and a set minimum size of eight reads. Chimeric sequences were excluded with the UCHIME3 module of vsearch. This included *de novo* chimera removal followed by reference-based chimera removal against the SILVA SSU 138 NR database (Bolyen et al., 2019; Quast et al., 2013), resulting in the final set of amplicon sequence variants (ASVs). Quality-filtered and primer-clipped sequences were mapped to ASVs by vsearch with a set identity of 0.97. Taxonomy assignments were performed with BLAST+ (blastn megablast, version 2.9.0) (Camacho et al., 2009) against the SILVA SSU 138 NR database with a minimum identity threshold of 90%. Additionally, we used identity and query coverage to mark uncertain blast hits. As recommended by the SILVA ribosomal RNA database project, we removed the taxonomic assignment for blast hits which did not meet the equation “(percent identity + query coverage)/2 ≤ 93” to obtain reliable taxonomic assignments. A phylogenetic tree was generated by aligning all sequences of the filtered dataset with MAFFT v7.407 (Katoh and Standley, 2013) at a maximum of 100 iterations. The tree was calculated using FastTree 2.1.7 (OpenMP) (Price et al., 2010) saved in newick format and midpoint rooted using FigTree (version 1.4.4) (Rambaut, 2018). Alpha-diversity indices and species richness were calculated with the ampvis2 package (version 2.5.9) (Andersen et al., 2018) and Faith’s phylogenetic diversity with picante (version 1.8.1) (Kembel et al., 2010) at same surveying effort (3,010 rarefied sequences per sample). Abundance bar charts, heatmap, and boxplots were created from ASVs with the ggplot2, ampvis2 package in RStudio^®^ v3.6.3 (R Core Team, 2017) with RStudio v1.1.456 (Wickham, 2016).

### Nucleotide Sequence Accession Numbers

Nucleotide gene sequences obtained from bacterial isolates in this study (see section “Isolation of Bacteria and Phylogenetic Affiliation”) were deposited in the GenBank^®^ nucleotide sequence database^3^ under the accession numbers MT254759 – MT254802. 16S rRNA gene amplicon sequences were submitted to the NCBI Sequence Read Archive^4^ (SRA) under the NCBI BioProject accession number PRJNA610782. The raw sequencing reads are available from the NCBI SRA under accession numbers SRR11250184 – SRR11250201. A detailed list of all accession codes is given in Supplementary Tables 3 and 4.

## Results and Discussion

### Bacterial Colonization of Different Materials and Inhibition of Growth by AGXX^®^ and GOX

The survival of cultivable, potentially pathogenic bacteria on the antimicrobial coated and uncoated sample materials was assessed after 1 (t1), 2 (t2), and 4 (t3) months exposure on board of the SIRIUS module and in the non-isolated control environment. The focus of this study was the occurrence of aerobic, heterotrophic bacteria.

**FIGURE 2:**
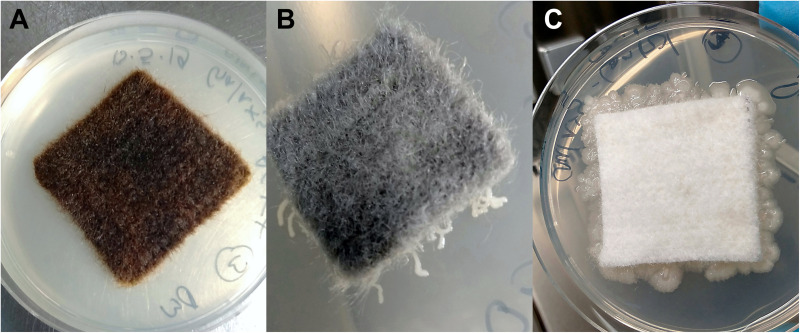
Cellulose fleece with and without antimicrobial coating after 48 h incubation at 37°C on R2A agar (following 2 months exposure): AGXX^®^-cellulose fleece **(A)**, GOX-cellulose fleece **(B)**, and uncoated cellulose fleece **(C)**.

Bacterial growth was inhibited in the presence of the antimicrobial coating AGXX^®^. R2A agar plates directly incubated with AGXX^®^ showed no bacterial growth regardless of exposure time (Figure 2A). Extensive bacterial growth occurred on uncoated control samples (Figure 2C). This supports evidence from earlier studies (Guridi et al., 2015; Landau et al., 2017; Loi et al., 2018; Vaishampayan et al., 2018; Sobisch et al., 2019) and demonstrates that the antimicrobial, growth-inhibiting properties of AGXX^®^ remain effective when applied to the new carrier materials.

To determine whether the new AGXX^®^-coated materials have a lasting, antibacterial effect, an additional cultivation approach was performed by imprinting the different materials from each time point onto R2A agar plates, allowing for transfer of adhering cells onto the agar. Without direct contact to the inhibiting material, bacterial colonies were recovered from all samples at all time points. This revealed that at least some bacteria can survive and resume growth upon removal of the contact catalyst AGXX^®^. The number of colony-forming units (CFUs) on agar plates following incubation revealed no notable reduction in viable cells by AGXX^®^ applied to polypropylene plastic or fibrous web polyester fleece. Counts of CFUs were not performed due to the often swarming, rapid growth of some bacterial isolates.

In contrast, a notable reduction of bacterial growth was observed for AGXX^®^ coating applied to cellulose fleece: out of six samples, four showed 50–70% growth reduction on AGXX^®^-cellulose agar imprints compared to uncoated cellulose control imprints (Supplementary Table 5). For this reason, only cellulose fleece was selected for further analysis (isolation and characterization of surviving bacteria and 16S rRNA gene amplicon analysis, see below). However, for two of the samples, the CFU for AGXX^®^ cellulose fleece was higher than for uncoated control fleece. These outliers may be explained by the low overall bacterial load (6–74 colonies per plate), suggesting cautious interpretation of low biomass data. Additionally, an important factor is the difference in cell recovery efficiency for the different materials and coatings. For example, spiking experiments with *B. subtilis* spores applied to the materials revealed that fewer cells are recovered from uncoated cellulose fleece (10–12% of applied cells) compared to the corresponding AGXX^®^-coated material (16–20%) (Ergün, 2019).

The novel, GO-based antimicrobial GOX, which was applied to cellulose fleece, “trapped” bacterial cells on its surface. We observed very localized bacterial growth, visible as microcolonies growing along the GOX-coated fibers that were in direct contact with the agar surface (Figure 2B). In contrast, uncoated cellulose fleece allowed extensive bacterial growth both underneath the sample material and on the surrounding agar (Figure 2C). The bacteria-immobilizing effect of GOX-cellulose fleece remained the same over the course of the 4 months incubation for both study sites.

### Bacterial Isolates Obtained From SIRIUS Module and Non-isolated Control Environment

To analyze which bacteria had survived on the antimicrobial materials, especially in terms of pathogenicity, bacterial colonies obtained from R2A agar plates imprinted with AGXX^®^-cellulose fleece, GOX-cellulose fleece and uncoated cellulose fleece after 1, 2, and 4 months exposure (see section “Bacterial Colonization of Different Materials and Inhibition of Growth by AGXX^®^ and GOX”) were purified and identified. Isolates belonging to Biosafety Level 2 (BSL-2) were characterized with regards to their antibiotic resistance profiles and ability to form biofilms.

The cultivated bacterial communities differed markedly between the SIRIUS module and the control environment. Both study sites share their high level of human traffic, but while the SIRIUS module is completely isolated, the control environment (microbiology laboratory) remains in steady contact with the outside environment through staff members coming and going. Notably, all isolates from both environments belonged to either the *Firmicutes*, *Actinobacteria* or *Proteobacteria*.

#### *Staphylococcus* spp. and *Bacillus* spp. Dominate Cultivated Bacterial Community in SIRIUS Module

Eighty bacterial isolates were recovered from coated and uncoated cellulose fleece materials across the three time-intervals and identified by MALDI-TOF biotyping and 16S rRNA gene sequencing. Most of the isolated species belong to the genera *Staphylococcus* (*n* = 43) and *Bacillus* (*n* = 10) (Table 1A). In addition, the following species were isolated: *Brevibacillus agri* (*n* = 2), *Paenibacillus vini* (*n* = 1), all of which belonging to the *Firmicutes*, *Corynebacterium mucifaciens* (*n* = 1), *Kocuria palustris* (*n* = 1), *Micrococcus luteus* (*n* = 8), *Rothia terrae* (*n* = 1), belonging to the *Actinobacteria*, *Moraxella osloensis* (*n* = 1) and *Roseomonas mucosa* (*n* = 1), the only two *Proteobacteria* (Table 1A). Noticeably, *M. luteus* was only isolated from uncoated cellulose fleece at the final time point and was not recovered from AGXX^®^- or GOX-cellulose.

**TABLE 1A:** Bacteria recovered from antimicrobial and uncoated surfaces in SIRIUS.

**Phylum**	**Genus**	**Species**	**Biosafety level**	**Cellulose-AGXX**	**Cellulose-GOX**	**Cellulose control**	**Total counts**
Firmicutes	***Aerococcus***	***viridans***	**2**	1	0	0	**1**
	*Bacillus*	*licheniformis*	1	1	1	1	**3**
	*Bacillus*	*simplex*	1	2	0	0	**2**
	*Bacillus*	*subtilis*	1	0	2	1	**3**
	*Bacillus*	sp.^1^	1	1	0	0	**1**
	*Bacillus*	sp.^2^	1	0	0	1	**1**
	*Brevibacillus*	*agri*	1	0	2	0	**2**
	*Paenibacillus*	*vini*	1	0	0	1	**1**
	*Staphylococcus*	*capitis*	1	1	0	0	**1**
	***Staphylococcus***	***caprae***	**2**	0	0	1	**1**
	***Staphylococcus***	***epidermidis***	**2**	6	2	0	**8**
	***Staphylococcus***	***haemolyticus***	**2**	3	8	0	**11**
	***Staphylococcus***	***hominis***	**2**	8	14	9	**31**
	*Staphylococcus*	*warneri*	1	0	0	1	**1**
Proteobacteria	***Moraxella***	***osloensis***	**2**	0	0	1	**1**
	***Roseomonas***	***mucosa***	**2**	1	0	0	**1**
Actinobacteria	***Corynebacterium***	***mucifaciens***	**2**	0	1	0	**1**
	*Kocuria*	*palustris*	1	0	1	0	**1**
	*Microccoccus*	*luteus*	1	5	3	0	**8**
	*Rothia*	*terrae*	1	0	0	1	**1**
				29	34	17	**80**

*Phylogenetic affiliation as assessed by MALDI-TOF-MS and 16S rRNA gene sequence analysis. The following isolates shared the same level of sequence identity with more than one species: ^1^*B. pumilus* (99.6%)/*B. safensis* (99.6%), ^2^*B. velezensis* (99.6%)/*B. siamensis* (99.6%). BSL-2 organisms are printed in bold.*

Out of 80 bacterial isolates, 55 belong to BSL-2 and are potential human pathogens (Table 1A). Almost all isolated BSL-2 organisms belong to the genus *Staphylococcus* (51 out of 55), namely *S. hominis* (*n* = 31), *S. haemolyticus* (*n* = 11), *S. epidermidis* (*n* = 8), and *S. caprae* (*n* = 1). The remaining BSL-2 organisms are *Aerococcus viridans*, *Corynebacterium mucifaciens*, *Roseomonas mucosa*, and *Moraxella osloensis* with one isolate each. While most of the *S. epidermidis* isolates were recovered from AGXX^®^-cellulose fleece (*n* = 6), most of the *S. haemolyticus* isolates originated from GOX-cellulose fleece (*n* = 9). Remarkably, no *S. epidermidis* or *S. haemolyticus* isolates were recovered from uncoated control samples. Meanwhile, *S. hominis*, the most abundant *Staphylococcus* species, was recovered from all three materials: (AGXX^®^-cellulose, *n* = 8; GOX-cellulose, *n* = 14; and uncoated cellulose fleece, *n* = 9). There was no noticeable tendency regarding the distribution of BSL-2 organisms in terms of antimicrobial coating or time points.

#### Diverse Bacterial Community Isolated From Control Environment

While the bacterial community isolated from SIRIUS-derived samples was strongly dominated by *Staphylococcus* spp. and *Bacillus* spp., the 103 isolates from the control environment were affiliated to 17 different genera within the *Firmicutes*, *Proteobacteria*, and *Actinobacteria* (Table 1B). *Micrococcus luteus* (*n* = 27) and *Roseomonas mucosa* (*n* = 12) were the most abundant species. Additionally, six *Bacillus* species (*n* = 12) were recovered while *Staphylococcus* species were much less abundant (*n* = 11) than on the SIRIUS module. The percentage of BSL-2 organisms (potential human pathogens) was significantly lower than on SIRIUS with 31 out of 103 isolates (30%). Most of the potential pathogens were *Proteobacteria*: *R. mucosa* (*n* = 12), *Paracoccus yeei* (*n* = 5), *Moraxella osloensis* (*n* = 4), and *Pantoea eucrina* (*n* = 2). Apart from one *Aerococcus viridans* isolate, all remaining BSL-2 organisms belonged to *Staphylococcus*: *S. haemolyticus* (*n* = 3), *S. hominis* (*n* = 3), and *S. epidermidis* (*n* = 1).

**TABLE 1B:** Bacteria recovered from antimicrobial and uncoated surfaces in the control environment.

**Phylum**	**Genus**	**Species**	**Biosafety level**	**Cellulose-AGXX**	**Cellulose-GOX**	**Cellulose-control**	**Total Counts**
Firmicutes	***Aerococcus***	***viridans***	**2**	0	0	1	**1**
	*Bacillus*	*firmus*	1	0	1	0	**1**
	*Bacillus*	*licheniformis*	1	1	1	2	**4**
	*Bacillus*	*megaterium*	1	0	1	1	**2**
	*Bacillus*	*pumilus*	1	0	0	1	**1**
	*Bacillus*	sp.^1^	1	1	1	0	**2**
	*Bacillus*	sp.^2^	1	0	0	2	**2**
	*Paenibacillus*	*lactis*	1	0	0	4	**4**
	*Staphylococcus*	*capitis*	1	0	4	0	**4**
	***Staphylococcus***	***epidermidis***	**2**	0	0	1	**1**
	***Staphylococcus***	***haemolyticus***	**2**	0	3	0	**3**
	***Staphylococcus***	***hominis***	**2**	3	0	0	**3**
Proteobacteria	*Massilia*	*timonae*	1	1	0	0	**1**
	***Moraxella***	***osloensis***	**2**	1	2	1	**4**
	***Pantoea***	***eucrina***	**2**	1	0	1	**2**
	*Paracoccus*	*contaminans*	1	1	0	1	**2**
	***Paracoccus***	***yeei***	**2**	2	2	1	**5**
	*Pseudomonas*	*stutzeri*	1	0	0	1	**1**
	***Roseomonas***	***mucosa***	**2**	6	1	5	**12**
	*Sphingomonas*	*desiccabilis*	1	0	1	0	**1**
Actinobacteria	*Dermacoccus*	*nishinomiyaensis*	1	3	0	0	**3**
	*Gordonia*	*rubripertincta*	1	1	1	0	**2**
	*Kocuria*	*rhizophila*	1	1	1	2	**4**
	*Kocuria*	*marina*	1	1	0	0	**1**
	*Micrococcus*	*luteus*	1	12	5	10	**27**
	*Pseudoarthrobacter*	*oxydans*	1	0	1	0	**1**
	*Streptomyces*	*thingirensis*	1	2	0	2	**4**
	*Streptomyces*	*canus*	1	0	0	1	**1**
	*Streptomyces*	sp.^3^	1	2	1	1	**4**
				38	26	39	**103**

*The following isolates shared the same level of sequence identity (≥99%) with more than one bacterial species: ^1^*B. stratosphericus*/*B. altitudinis*/*B. aerophilus.*^2^*B. velezensis*/*B. siamensis*. ^3^*S. aureorectus*/*S. calvus*/*S. asterosporus*. BSL-2 organisms are printed in bold.*

#### Prevalence of Human Commensal Bacteria in Confined Habitats

Dominance of human-derived bacteria is expected in confined, human-made habitats with no access to the natural environment. All *Staphylococcus* species isolated on the SIRIUS module (*S. hominis*, *S. haemolyticus*, *S. epidermidis*, *S. capitis*, *S. caprae*, and *S. warneri*) are commonly associated with the human skin and mucosal flora. Since many of these bacteria can act as opportunistic human pathogens, the prevalence of BSL-2 organisms within SIRIUS (69% of isolates) was accordingly higher than in the control environment (30% of isolates). Indeed, over 92% of BSL-2 organisms isolated from SIRIUS samples belonged to the *Staphylococci.* The high abundance of *Staphylococcus* and *Bacillus* isolates inside SIRIUS is in accordance with observations by Schwendner et al. (2017), who carried out an extensive survey of the microbial community during the Mars500 project, the first simulated space mission taking place inside the SIRIUS module over 17 months. They found that *Staphylococcus* spp. strongly dominated the airborne cultivable community. By comparison, the microbial community on surfaces showed a greater diversity and was dominated by both *Bacillus* spp. and *Staphylococcus* spp. alongside less abundant genera from the *Proteobacteria* and *Actinobacteria*.

Prevalence of *Staphylococcus* and *Bacillus* species has also been reported by numerous cultivation-based surveys of the microbial community on the ISS (Novikova et al., 2006; Checinska et al., 2015; Sobisch et al., 2019). No serious harmful pathogens such as MRSA were detected in any of our samples. Furthermore, in contrast to the ISS surveys, we detected no enterococci, enterobacteria or corynebacteria.

### Antibiotic Resistance and Biofilm Formation of Isolates

Potential human pathogens were characterized in terms of biofilm formation and antibiotic resistance. Disk diffusion assays revealed resistance to at least one antibiotic in all 25 tested BSL-2 organisms isolated from SIRIUS samples. Most were multiresistant (resistant to antimicrobial agents from three or more categories Table 2A). Resistance against erythromycin, kanamycin, and ampicillin was common amongst *Staphylococcus* isolates. Of all *Staphylococcus* isolates, 78% were resistant to erythromycin, 70% to kanamycin, and 48% to ampicillin (Table 2A). Antibiotic resistance profiles showed considerable variation between isolates, including strains of the same species derived from the same material and time point. The isolate with the highest level of antibiotic resistance (*S. haemolyticus* GAM2-7, isolated from AGXX^®^ – cellulose fleece) was resistant to eight out of the 12 antibiotics tested: ampicillin, ciprofloxacin, clindamycin, erythromycin, kanamycin, oxacillin, tetracycline, and tigecycline. The abundance of resistances among isolates did not change over time (1 to 4 months).

**TABLE 2A:** Antibiotic resistant and biofilm forming BSL-2 isolates from SIRIUS.

**Number**	**Name**	**Species**	**Biofilm formation**	**Antibiotic resistance**
1	GAM1-25	*A. viridans*	^A^	NIT
2	GCM1-14	*M. osloensis*	^A^	ERY, MEM, NAL
3	GXM3-1b	*S. caprae*	+++	ERY
4	GAM1-42	*S. epidermidis*	++	ERY, KAN, TET^B^
5	GAM1-43	*S. epidermidis*	+++	ERY, KAN
6	GXM2-7	*S. epidermidis*	++	AMP, ERY, KAN, OXA, OFX, TET
7	GCM1-15	*S. haemolyticus*	++	ERY, KAN, TET
8	GAM2-7	*S. haemolyticus*	++	AMP, CIP, CLI^B^, ERY^B^, KAN, OXA, TET, TGC
9	GAM3-3a	*S. haemolyticus*	++	KAN, NIT, NOR, OFX, OXA
10	GAM3-5a	*S. haemolyticus*	++	ERY, KAN, OXA
11	GXM2-4a	*S. haemolyticus*	++	CLI, ERY, OXA, OFX, TGC
12	GXM3-8b	*S. haemolyticus*	++	AMP, ERY, KAN, OXA, TET
13	GCM1-57	*S. hominis*	+++	AMP^B^, ERY
14	GCM1-53	*S. hominis*	++	AMP, ERY
15	GCM1-59	*S. hominis*	+++	AMP, ERY
16	GCM3-1a	*S. hominis*	+	AMP, CIP, ERY, KAN, TET
17	GAM1-24	*S. hominis*	+	ERY, KAN, OXA, TET^B^
18	GAM2-5	*S. hominis*	+++	ERY, KAN, TET
19	GAM3-2	*S. hominis*	++	ERY
20	GXM1-1c	*S. hominis*	+++	KAN, OFX
21	GXM1-8	*S. hominis*	+	GEN, KAN, OFX
22	GXM2-2	*S. hominis*	+	AMP
23	GXM3-1a	*S. hominis*	+	AMP, CLI*, ERY, KAN, NIT
24	GXM3-2	*S. hominis*	++	AMP, KAN
25	GCM1-63	*S. warneri*	+++	AMP, ERY, KAN, OFX, OXA

*+++, strong biofilm formation; ++, moderate biofilm formation; +, weak biofilm formation; –, no biofilm formation; AMP, ampicillin, 2 μg; CIP, ciprofloxacin, 5 μg; CLI, clindamycin, 2 μg; ERY, erythromycin, 15 μg; GEN, gentamicin, 10 μg; KAN, kanamycin, 30 μg; MEM, meropenem, 10 μg; OXA, oxacillin, 1 μg, OFX, ofloxacine, 5 μg; NAL, nalidixic acid, 30 μg; TGC, tigecycline, 15 μg; TET, tetracycline 30 μg; NOR, norfloxacin, 10 μg; NIT, nitrofurantoin, 100 μg. ^*A*^Biofilm formation for these isolates was not analyzed as growth rates in standard media were too low to follow the standard protocol. ^*B*^Inhibition zone diameters for these antibiotics were on the border between resistant and susceptible. Concentrations are μg per disk. Strain denominations: GCM1, Galaxy, Control, Moscow, t1; GAM1, Galaxy, AGXX, Moscow, t1; GXM1, Galaxy, GOX, Moscow, t1; GCM2, Galaxy, Control, Moscow, t2; GAM2, Galaxy, AGXX, Moscow, t2; GXM2, Galaxy, GOX, Moscow, t2; GCM3, Galaxy, Control, Moscow, t3; GAM3, Galaxy, AGXX, Moscow, t3; GXM3; Galaxy, GOX, Moscow, t3.*

Staphylococcal resistance against erythromycin and ampicillin was also abundant amongst isolates retrieved from AGXX^®^ samples exposed on the ISS (Sobisch et al., 2019), while resistance against kanamycin (detected in 70% of *Staphylococcus* spp. from SIRIUS) was rare in coagulase negative Staphylococci from the ISS (Schiwon et al., 2013; Sobisch et al., 2019). Notably, BSL-2 isolates from the non-isolated control environment showed significantly fewer resistances, with many isolates being sensitive to all tested antibiotics (Table 2B). Out of 23 isolates, nine showed no resistance against any of the antibiotics tested. Moreover, the frequency of erythromycin and kanamycin resistance among *Staphylococcus* spp. was considerably lower (Table 2B). This may be explained by several facts: (i) isolates from the non-confined environment comprised many environmental organisms in contrast to human inhabitants, (ii) the microbiome of individuals differs considerably according to their exposure to antibiotics, and (iii) under confined conditions, bacteria in the SIRIUS module lack competition with microorganisms from the natural environment.

**TABLE 2B:** Antibiotic resistance and biofilm formation of BSL-2 isolates from control environment.

**Number**	**Name**	**Species**	**Biofilm formation**	**Antibiotic resistance**
1	GCB1-10	*A. viridans*	^A^	NIT, MEM, NOR
2	GAB1-9	*M. osloensis*	^A^	/
3	GXB2-1a	*M. osloensis*	^A^	ERY, NAL
4	GCB1-14	*M. osloensis*	^A^	ERY, MEM, NAL, TET
5	GXB2-2	*P. yeei*	++	/
6	GXB3-2c	*P. yeei*	+++	CIP, GEN
7	GCB3-1b	*P. yeei*	*++*	/
8	GCB3-1d	*P. yeei*	*++*	/
9	GCB2-6	*R. mucosa*	^A^	MEM
11	GAB2-6	*R. mucosa*	^A^	MEM
12	GAB2-7	*R. mucosa*	^A^	/
14	GAB3-2b	*R. mucosa*	^A^	/
15	GCB1-9b	*R. mucosa*	^A^	/
16	GCB3-2a3	*R. mucosa*	^A^	/
17	GXB1-8	*S. capitis*^B^	++	AMP, OXA, OFX
18	GCB1-13	*S. epidermidis*	++	AMP, ERY
19	GXB1-10	*S. haemolyticus*	++	GEN, KAN
20	GXB1-6	*S. haemolyticus*	++	AMP, OXA
21	GAB3-1h	*S. hominis*	+	AMP
22	GAB2-2	*S. hominis*	+++	AMP, ERY
23	GAB2-3	*S. hominis*	+	/

*+++, strong biofilm formation; ++, moderate biofilm formation; +, weak biofilm formation; –, no biofilm formation; AMP, ampicillin, 2 μg; CIP, ciprofloxacin, 5 μg; CLI, clindamycin, 2 μg; ERY, erythromycin, 15 μg; GEN, gentamicin, 10 μg; KAN, kanamycin, 30 μg; MEM, meropenem, 10 μg; OXA, oxacillin, 1 μg, OFX, ofloxacine, 5 μg; NAL, nalidixic acid, 30 μg; TGC, tigecycline, 15 μg; TET, tetracycline 30 μg; NOR, norfloxacin, 10 μg; NIT, nitrofurantoin, 100 μg. /, no resistance detected. Concentrations are μg per disk. ^*A*^Biofilm formation for these isolates was not analyzed as growth rates on standard media were too low to follow the standard protocol. ^*B*^*S. capitis* is a BSL-1 organism; 16S rRNA gene sequence analysis for this isolate was inconclusive with the same level of sequence identity to *S. capitis* and *S. caprae* (BSL-2). Therefore, the isolate was included. Additional MALDI-TOF MS analysis, however, identified the isolate as *S. capitis*. Strain denominations: GCB1, Galaxy, Control, Berlin, t1; GAB1, Galaxy, AGXX, Berlin, t1; GXB1, Galaxy, GOX, Berlin, t1; GCB2, Galaxy, Control, Berlin, t2; GAB2, Galaxy, AGXX, Berlin, t2; GXB2, Galaxy, GOX, Berlin, t2; GCB3, Galaxy, Control, Berlin, t3; GAB3, Galaxy, AGXX, Berlin, t3; GXB3; Galaxy, GOX, Berlin, t3.*

In addition to testing for antibiotic resistance, potential human pathogens were screened for their ability to form biofilms. Only those isolates that showed sufficiently fast growth under the conditions specified in the protocol by Vaishampayan et al. (2018) were tested (Tables 2A,B). All tested BSL-2 isolates from the fleece materials exposed on SIRIUS and the control environment were capable of biofilm formation. Out of 23 SIRIUS isolates (all *Staphylococcus* spp.), seven showed strong, 11 moderate, and five weak biofilm formation. Out of 11 tested isolates from the control environment (*Paracoccus yeei*, *Staphylococcus* spp.), two showed strong, seven moderate, and two weak biofilm formation. In both environments, strong and weak biofilm formers were isolated from all three materials and time points with no visible correlation between biofilm formation and level of antibiotic resistance.

The ability to form biofilms could provide competitive advantages over other airborne bacteria. In contrast to Sobisch et al. (2019) correlation between antimicrobial coating and frequency or degree of antibiotic resistance was not detected. This effect may be more pronounced after longer exposure times. However, the isolate with the highest number of antibiotic resistances (resistance against 8 of 12 antibiotics tested) was a strain of *S. haemolyticus* retrieved from AGXX^®^-coated fleece.

### Bacterial Community Composition – Similar Taxa Occur in Both Environments

The growth conditions applied in this study were chosen to select for common human pathogens, and targeted aerobic, heterotrophic bacteria. To supplement cultivation-based results and identify potential pathogens not detected by isolation, the bacterial communities present on the three materials after 1, 2, and 4 months exposure were examined by analysis of 16S rRNA gene amplicons from DNA extracted from fleece samples.

**FIGURE 3:**
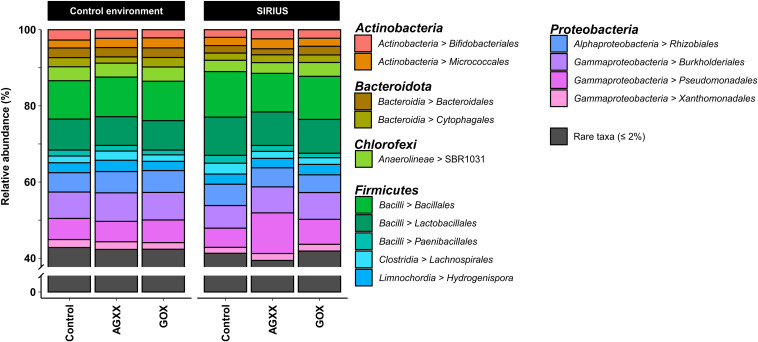
Relative abundance of bacterial orders from antimicrobial and uncoated cellulose fleece exposed in SIRIUS and in non-isolated control environment detected by amplicon sequencing. Reads from time points t1, t2, and t3 were condensed for each sample in this illustration.

**FIGURE 4:**
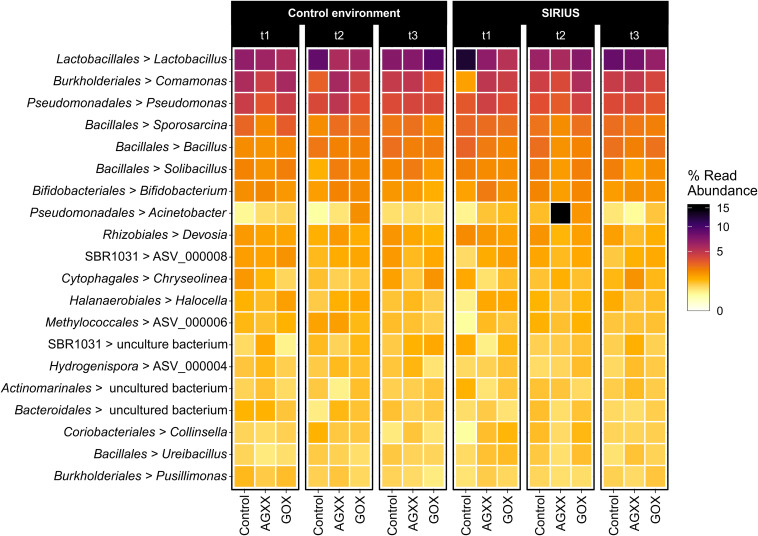
Heatmap showing the top 20 dominant bacterial genera from antimicrobial and uncoated fleece samples exposed in SIRIUS and the non-confined control environment at different timepoints detected by amplicon sequencing. Abbreviations: t1, time point 1, 1 month; t2, time point 2, 2 months; t3, time point 3, 4 months.

While culture-based communities differed considerably between SIRIUS and the control environment, amplicon sequence data suggest that the bacterial community composition of the two environments is highly similar (Figures 3, 4). The dominant phyla in both sites were *Firmicutes* (34–47% of the total bacterial community), *Proteobacteria* (22–39%), and *Actinobacteria* (10–14%). Together, *Firmicutes*, *Proteobacteria*, and *Actinobacteria* constituted over 90% of the communities in all the samples. Additional dominant phyla not recovered by cultivation studies were *Bacteroidetes* (6–9%) and *Chloroflexi* (5–7%).

The majority of *Firmicutes* belonged to the class *Bacilli* (19–29% of total bacterial population), followed by *Clostridia* (7–9%) and *Limnochordia* (4–6%). While more than half of all SIRIUS isolates belonged to *Staphylococcus* spp. (Tables 1A,B), *Staphylococcales* only constituted 0.1–0.6% of total sequences. Instead, *Bacillales* (9–12%) and *Lactobacillales* (6–13%) were the dominant orders within the *Bacilli* based on amplicon sequence data (Figure 3). Within the *Actinobacteria*, *Micrococcales* (2–2.5% of total sequences), *Corynebacteriales* (2–2.5%), and *Bifidobacteriales* (2–2.5%) were the dominant orders (Figure 3). Proteobacterial sequences mostly belonged to the *Pseudomonadales* (6–19%), *Burkholderiales* (7–9%), and *Rhizobiales* (5–6%) (Figure 3). At genus level, the dominant taxa were *Lactobacillus* (6–13% of total reads), *Pseudomonas* (4–5%), *Comamonas* (2–6%), *Bacillus* (2–3%), *Sporosarcina* (2–3%), *Bifidobacterium* (2–2.5%), *Solibacillus* (1.5–2.5%), *Devosia* (1.5–2%), and *Acinetobacter* (0.5–2%) (Figure 4). *Moraxella* and *Roseomonas* (the only *Proteobacteria* isolated from SIRIUS) made up less than 0.05% of amplicon sequences. There was no correlation between the distribution of the different taxa and exposure time or antimicrobial coating (Figure 4).

**FIGURE 5:**
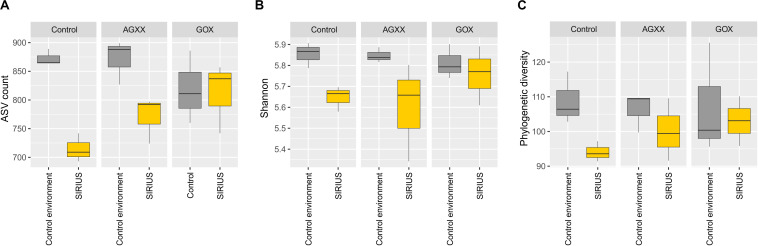
Boxplots of alpha-diversity indices of all samples summarized by time. **(A)** Observed ASVs, **(B)** Shannon index, **(C)** Faiths phylogenetic diversity index. Comparisons were performed at same sequencing effort (rarefied selection of 3,010 reads per sample) and phylogenetic diversity (Faith’s PD) was calculated on a midpoint-rooted phylogenetic tree of all ASVs.

The relative abundance of the dominant bacterial taxa was highly similar between the two sites (Figures 3, 4), but analysis of the alpha-diversity revealed that the overall bacterial diversity was lower in SIRIUS than in the non-confined control environment (Figure 5).

In summary, the same taxa appear to dominate both the SIRIUS module and the control environment, even though the alpha-diversity was lower in SIRIUS. The community composition was not affected by the antimicrobial materials during the time course of the study. Opportunistic, human commensal pathogens identified by isolation do not appear to dominate the bacterial communities within SIRIUS.

### Comparison of the SIRIUS Microbiome to Microbial Communities in Similar Confined Habitats

Prevalence of *Firmicutes*, *Proteobacteria*, and *Actinobacteria* has been reported almost uniformly by both cultivation-dependent and molecular surveys of the ISS microbiome (Novikova et al., 2006; Checinska et al., 2015; Singh et al., 2018; Sobisch et al., 2019; Voorhies et al., 2019). The bacterial community inside the NEK facility during the MARS500 project revealed the same phyla using DNA microarrays and next generation sequencing (Schwendner et al., 2017). However, when comparing our results to those of Schwendner et al. (2017) at class and genus level, there are pronounced differences in the bacterial communities from SIRIUS19 and MARS500 samples. Strikingly, there is a much higher percentage of potential, opportunistic pathogens in the bacterial communities reported by Schwendner et al. (2017).

*Proteobacteria* rather than *Firmicutes* were most abundant in the study by Schwendner et al., with *Ralstonia*, *Acinetobacter*, *Pseudomonas*, *Burkholderia*, and *Moraxella* being the major genera. Our proteobacterial sequences on the other hand mostly belonged to *Comamonas* and *Pseudomonas* (Figure 4, Supplementary Figure 1). *Acinetobacter* was less abundant; *Ralstonia*, *Burkholderia*, and *Moraxella* constituted less than 0.05% of sequences, even though *Moraxella* was identified by isolation. While over 60% of *Firmicutes* sequences from SIRIUS belonged to the *Bacilli* (19–27% of total reads) and only 20% to *Clostridia* (6–10% of total reads), two thirds of the *Firmicutes* in the study by Schwendner et al. (2017) were represented by *Clostridia* (over 22% of total reads). In contrast to our findings, *Staphylococcus* was one of the dominant genera within the *Firmicutes* (6% compared to 0.1–0.4% of SIRIUS sequences) alongside *Streptococcus* (4.8% compared to 0.2%). In contrast, *Firmicutes* sequences from SIRIUS samples were dominated by *Lactobacillus* (6–13% of total reads), *Bacillus* (2–3%), *Sporosarcina* (2–3%), and *Solibacillus* (1.5–2.5%). The composition of the microbial community within the NEK facility differs strongly from one study to the next which is potentially linked to a changing crew with individual microbiomes.

### Bacterial Community Composition on SIRIUS Remains Stable Throughout 4-Months Study

The microbial community composition on our sample materials exposed inside SIRIUS did not change over time in terms of the dominant taxa (Figures 3, 4). Sobisch et al. (2019) reported an increase in microbial diversity on AGXX^®^ samples exposed on the ISS after 12 and 19 months, with appearance of additional genera like *Enterococcus* and *Pseudomonas* at later timepoints. Voorhies et al. (2019) observed a decrease in *Proteobacteria* over time with a simultaneous increase in *Firmicutes*, *Bacteroidetes*, and *Actinobacteria* in ISS crew members’ skin microbiomes during a long-term space mission. This suggests that competition between these groups is affected by spaceflight. In a microbial succession study in an inflated lunar/Mars analog habitat, Mayer et al. (2016) observed a similar shift from predominantly *Proteobacteria* and *Firmicutes* (especially *Bacillaceae*) to other members of *Firmicutes* (*Clostridiales*) and *Actinobacteria* (especially *Corynebacteriaceae*) following human occupation. By comparison, Schwendner et al. (2017) found strong fluctuations in the microbial community within SIRIUS during the Mars500 project without any directional trend. They attributed this to the unequal distribution of bacteria in air. Our molecular data meanwhile revealed no directional shift of the detected taxa over time.

### Outlook

As part of the next SIRIUS isolation experiment (scheduled to start in autumn 2020 over a period of 8 months), we plan to carry out further studies on the long-term antimicrobial effect of AGXX^®^ and GOX. The longer time frame will give an enhanced insight into the effect of the two antimicrobials on survival and composition of the bacterial community on surfaces inside the artificial space station. Improving the methodology to recover microbial cells from the sample materials will allow for thorough assessment of bacterial survival rates and the identity of viable bacteria, as well as provide increased DNA yields. This will enable us to assess not only the microbial composition based on 16S rRNA gene amplicons, but rather study the whole metagenome including virulence and resistance factors in order to reveal the survival strategies of microorganisms surviving on the antimicrobial materials.

## Data Availability Statement

The sequence data that support the findings of this study are openly available in GenBank^®^ and the NCBI Sequence Read Archive under accession codes detailed in Supplementary Tables 3 and 4.

## Author Contributions

EG and DW designed the project and supervised all the experiments. DW, DS, AP, FH, HO, JM, and SK performed the experiments and analyzed the data. RA designed and prepared the GOX materials. DW, AP, DS, SK, and EG wrote the manuscript and designed the figures and tables. NN provided us access to the facilities at the IMBP in Moscow, Russia. RD, NN, RH, and OW contributed with insightful discussions on the experimental design. All authors interpreted the results, read, and revised the manuscript. All authors contributed to the article and approved the submitted version.

## Conflict of Interest

The authors declare that the research was conducted in the absence of any commercial or financial relationships that could be construed as a potential conflict of interest.

## Footnotes

1 https://lpsn.dsmz.de/

2 http://www.eucast.org

3 https://www.ncbi.nlm.nih.gov/genbank/

4 https://www.ncbi.nlm.nih.gov/sra
