# Supplementary material for: Novel Antimicrobial Cellulose Fleece Inhibits Growth of Human-Derived Biofilm-Forming Staphylococci During the SIRIUS19 Simulated Space Mission
Source: Front Microbiol. 2020 Jul 29;11:1626. doi: 10.3389/fmicb.2020.01626 (PMC7405646; doi:10.3389/fmicb.2020.01626)
Supplement: Supplementary file 1 [file Data_Sheet_1.docx]

**Supplementary Data**

**Novel antimicrobial cellulose fleece inhibits growth of human-derived biofilm-forming Staphylococci during the SIRIUS19 simulated space mission**

**Running title: Antimicrobial coated cellulose inhibits Staphylococci**

Wischer, D.^1^, Schneider, D.^2^, Poehlein, A.^2^, Herrmann F.^1^, Oruc, H^1^, Meinhardt, J.^1^, Wagner, O.^3^, Ahmed, R. ^3^, Kharin, S.^4^, Novikova, N.^4^, Haag, R.^3^, Daniel, R.^2^, Grohmann, E.^1*^

^1^Beuth University of Applied Sciences, Life Sciences and Technology, Microbiology, Seestrasse 64, 13347 Berlin, Germany

^2^Georg-August-University Göttingen, Institute of Microbiology and Genetics, Department of Genomic and Applied Microbiology & Göttingen Genomics Laboratory, Grisebachstr. 8, D-37077, Göttingen, Germany

^3^Freie Universität Berlin, Institute for Chemistry and Biochemistry, Takustrasse 3, 14195 Berlin, Germany

^4^Institute of Biomedical Problems (IBMP), Khoroshevskoye Shosse 76 A, 123007, Moscow, Russia

*** Correspondence:**

Corresponding Author

[Elisabeth.grohmann@beuth-hochschule.de](mailto:Elisabeth.grohmann@beuth-hochschule.de)

Keywords: antimicrobial material, SIRIUS, isolated environment_,_ space microbiology, antibiotic resistance, human-commensal bacteria

**Supplementary Table 1.** Oligonucleotides used for amplification of bacterial 16S rRNA genes.

| Primer set | Sequence  (5´🡪 3`) | Amplicon  size [bp] | Annealing  temp. [C°] | Reference |
| --- | --- | --- | --- | --- |
| 27_fwd  536_rev | AGAGTTTGATCATGGCTCAG GTATTACCGCGGCTGCTG | ~ 509 | 50 | Lane *et al.*, 1991 Leser *et al.*, 2002 Weisburg *et al.*, 1991 |
| 341_fwd 805_rev | CCTACGGGNGGCWGCAG GGACTACHVGGGTWTCTAAT | ~ 464 | 50 | Herlemann *et al.*, 2011 Herlemann *et al.*, 2011 |
| S-D-Bact-0341-b-S-17-N | TCGTCGGCAGCGTCAGATGT TATAAGAGACAGCCTACGGG GGCWGCAG^1, 2^ | ~464 | 57 | Klindworth *et al.*, 2013 |
| S-D-Bact-0785-a-A-21-N | GTCTCGTGGGCTCGGAGATGTGTATAAGAGACAGGACTACHVGGGTATCTAATCC^1, 3^ |  |  | Klindworth *et al.*, 2013 |

N, any base; W, A or T; H, A or C or T; V, A or C or G. ^1^ Underlined parts of primer sequences denote Illumina adapter sequences; remainder of primer sequence is based on ^2^ 341_fwd and ^3^ 805_rev.

**Supplementary Table 2.** Antibiotic disc diffusion method.

|  |  |  | ***Staphylococcus* spp.** | ***Moraxella osloensis*** | ***Roseomonas mucosa ^A)^*** | ***Aerococcus  viridans*** | ***Paracoccus  yeei ^A)^*** |
| --- | --- | --- | --- | --- | --- | --- | --- |
| **Antibiotic class** | **Antibiotic (Abbreviation)** | | **Content (μg)** | **Content (μg)** | **Content (μg)** | **Content (μg)** | **Content (μg)** |
| **Penicillins:** | Ampicillin | (AMP) | 2 | - | - | 2 | - |
|  | Oxacillin | (OXA) | 1 | - | - | - | - |
| **Fluoroquinolones:** | Ciprofloxacin | (CIP) | 5 | ^B)^ | 5 | 5 | 5 |
|  | Ofloxacin | (OFL)) | 5 | ^B)^ | - | - | - |
|  | Norfloxacin | (NOR) | 10 | - | - | 10 | - |
|  | Nalidixic Acid | (NAL) | - | 30 | - | - | - |
| **Carbapenems:** | Imipenem | (IMI) | - | 10 | - | - | - |
|  | Meropenem | (MEM) | - | 10 | 10 | 10 | 10 |
| **Aminoglycosides:** | Gentamicin | (GEN) | 10 | - | 10 | - | 10 |
|  | Kanamycin | (KAN) | 30 | - | - | - | - |
| **Tetracyclines:** | Tetracycline | (TET) | 30 | 30 | - | - | - |
|  | Tigecycline | (TGC) | 15 | - | - | - | - |
| **Macrolides, Lincosamides:** | Clindamycin | (CLI) | 2 | - | - | - | - |
|  | Erythromycin | (ERY) | 15 | 15 | - | - | - |
| **Glycopeptides:** | Vancomycin | (VAN) | - | - | - | 5 | - |
| **Miscellaneous:** | Nitrofurantoin | (NIT) | 100 | - | - | 100 | - |

Antibiotic selection, disc contents and inhibition zones (not shown) according to EUCAST (http://www.eucast.org).
"-" indicates that susceptibility testing is not recommended by EUCAST as the organism is a poor target for therapy with the agent. ^A)^ Susceptibility testing of *R. mucosa* and *P. yeei* followed criteria for *Pseudomonas* spp. ^B)^ The nalidixic acid disk diffusion test can be used to screen for fluoroquinolone resistance. Isolates categorised as susceptible to nalidixic acid can be reported susceptible to ciprofloxacin and ofloxacin.

| **Supplementary Table 3.** Accession codes for 16S rRNA gene sequences from bacterial isolates  SUB7110426 GCB1_10_A_viridans        MT254759  SUB7110426 GAM1_25_A_viridans        MT254760  SUB7110426 GCB3_1a1a_P_lactis        MT254761  SUB7110426 GCB3_1e_P_lactis          MT254762  SUB7110426 GXB1_8_S_capitis          MT254763  SUB7110426 GAM3_5a_S_haemolyticus     MT254764  SUB7110426 GXM3_8b_S_haemolyticus     MT254765  SUB7110426 GAM3_2_S_hominis          MT254766  SUB7110426 GAB2_3_S_hominis          MT254767  SUB7110426 GAM2_2_S_hominis          MT254768  SUB7110426 GXM2_2_S_hominis          MT254769  SUB7110426 GAM1_28a_B_simplex        MT254770  SUB7110426 GXB1_10a_Bacillus          MT254771  SUB7110426 GXB1-10e_B_firmus          MT254772  SUB7110426 GCB2_7_Bacillus            MT254773  SUB7110426 GAM1_31_Bacillus          MT254774  SUB7110426 GAB1_4_Bacillus            MT254775  SUB7110426 GAB2_5a_B_licheniformis    MT254776  SUB7110426 GCM2_3a_Bacillus          MT254777  SUB7110426 GCB2_6a_B_licheniformis    MT254778  SUB7110426 GCB1_16a_B_velezensis      MT254779  SUB7110426 GCM2_3b_B_velezensis      MT254780  SUB7110426 GXM2_1b_B_subtilis        MT254781  SUB7110426 GXM3_11_C_mucifaciens      MT254782  SUB7110426 GCB3_1eS_S_canus          MT254783  SUB7110426 GCB3_w_S_thingirensis      MT254784  SUB7110426 GAB3_1e_S_thingirensis     MT254785  SUB7110426 GCB1_2_K_rhizophila        MT254786  SUB7110426 GCB3_y_K_rhizophila        MT254787  SUB7110426 GAB1_3y_M_luteus          MT254788  SUB7110426 GAM3_4_M_luteus            MT254789  SUB7110426 GAB2_1_R_mucosa            MT254790  SUB7110426 GAB3_1d_M_timonae          MT254791  SUB7110426 GAB2_7_R_mucosa            MT254792  SUB7110426 GXB1_5_R_mucosa            MT254793  SUB7110426 GAB2_4_R_mucosa            MT254794  SUB7110426 GXB1_5s_S_desiccabilis     MT254795  SUB7110426 GXB2_2_P_yeei              MT254796  SUB7110426 GAB3_1t1_P_yeei            MT254797  SUB7110426 GXB3_2c_P_yeei            MT254798  SUB7110426 GAB1_4_Paracoccus          MT254799  SUB7110426 GXB2_1a_M_osloensis        MT254800  SUB7110426 GCB2_2_P_eucrina          MT254801  SUB7110426 GCB3_2c1_P_stutzeri        MT254802  **Supplementary Table 4.** Accession codes for 16S rRNA gene amplicon sequences   \| study SRA: SRP251836  BioProject: PRJNA610782  BioSample \| Sample \| SRA Accession \| \| --- \| --- \| --- \| \| SAMN14313857 \| 1904Bt1-AGXX \| SRR11250201 \| \| SAMN14313858 \| 1904Bt1-GOX \| SRR11250200 \| \| SAMN14313859 \| 1904Bt1-C \| SRR11250199 \| \| SAMN14313860 \| 1905Bt2-AGXX \| SRR11250198 \| \| SAMN14313861 \| 1905Bt2-GOX \| SRR11250197 \| \| SAMN14313862 \| 1905Bt2-C \| SRR11250196 \| \| SAMN14313863 \| 1907Bt3-AGXX \| SRR11250195 \| \| SAMN14313864 \| 1907Bt3-GOX \| SRR11250194 \| \| SAMN14313865 \| 1907Bt3-C \| SRR11250193 \| \| SAMN14313866 \| 1904Mt1-AGXX \| SRR11250192 \| \| SAMN14313867 \| 1904Mt1-GOX \| SRR11250191 \| \| SAMN14313868 \| 1904Mt1-C \| SRR11250190 \| \| SAMN14313869 \| 1905Mt2-AGXX \| SRR11250189 \| \| SAMN14313870 \| 1905Mt2-GOX \| SRR11250188 \| \| SAMN14313871 \| 1905Mt2-C \| SRR11250187 \| \| SAMN14313872 \| 1907Mt3-AGXX \| SRR11250186 \| \| SAMN14313873 \| 1907Mt3-GOX \| SRR11250185 \| \| SAMN14313874 \| 1907Mt3-C \| SRR11250184 \|   **Supplementary Table 5.** CFUs recorded on R2A plates imprinted with coated and uncoated cellulose fleece at different time points following 48h incubation at 37°C   \| \| SIRIUS module \| \| \| \| \| --- \| --- \| --- \| --- \| \|  \| AGXX® \| GOX \| Control \| \| 1 month \| 28 \| 16 \| 10 \| \| 2 months \| 6 \| 10 \| 12 \| \| 4 months \| 8 \| 15 \| 12 \| \| Total \| 42 \| 41 \| 34 \| \| Control environment \| \| \| \| \|  \| AGXX® \| GOX \| Control \| \| 1 month \| 20 \| 14 \| 74 \| \| 2 months \| 7 \| 5 \| 15 \| \| 4 months \| 70 \| 30 \| 50 \| \|  \| 97 \| 49 \| 139 \| \|  \|  \|  \|  \| \|  \|  \|  \| \| --- \| --- \| --- \| --- \| --- \| --- \| --- \| --- \| --- \| --- \| --- \| --- \| --- \| --- \| --- \| --- \| --- \| --- \| --- \| --- \| --- \| --- \| --- \| --- \| --- \| --- \| --- \| --- \| --- \| --- \| --- \| --- \| --- \| --- \| --- \| --- \| --- \| --- \| --- \| --- \| --- \| --- \| --- \| --- \| --- \| --- \| --- \| --- \| --- \| --- \| --- \| --- \| --- \| --- \| --- \| --- \| | |  |  |
| --- | --- | --- | --- | --- | --- | --- | --- | --- | --- | --- | --- | --- | --- | --- | --- | --- | --- | --- | --- | --- | --- | --- | --- | --- | --- | --- | --- | --- | --- | --- | --- | --- | --- | --- | --- | --- | --- | --- | --- | --- | --- | --- | --- | --- | --- | --- | --- | --- | --- | --- | --- | --- | --- | --- | --- | --- | --- | --- | --- | --- | --- | --- | --- | --- | --- | --- | --- | --- | --- | --- | --- | --- | --- | --- | --- | --- | --- | --- | --- | --- | --- | --- | --- | --- | --- | --- | --- | --- | --- | --- | --- | --- | --- | --- | --- | --- | --- | --- | --- | --- | --- | --- | --- | --- | --- | --- | --- | --- | --- | --- | --- | --- | --- | --- | --- | --- |
|  |  |  |  |

**
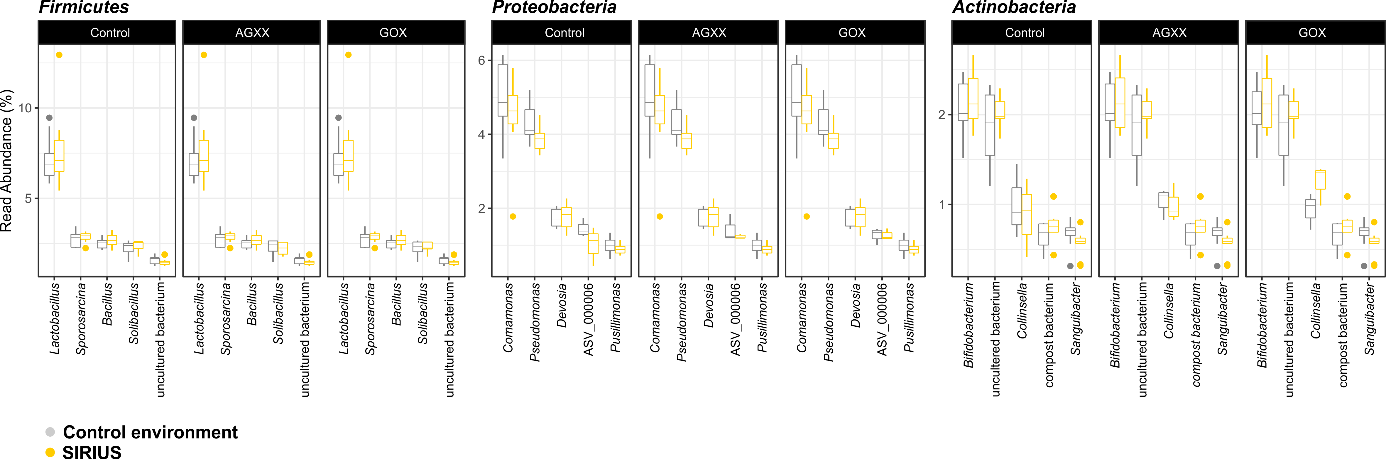
**

**Supplementary Figure 1. Boxplots showing dominant bacterial genera of the three dominant phyla detected on antimicrobial and uncoated fleece samples exposed inside SIRIUS module and non-isolated control environment.**

**References**

Klindworth, A., Pruesse, E., Schweer, T., Peplies, J., Quast, C., Horn, M., *et al*. (2013). Evaluation of general 16S ribosomal RNA gene PCR primers for classical and next-generation sequencing-based diversity studies. *Nucleic Acids Res*. 41:e1. doi:10.1093/nar/gks808.

Lane, D.J. (1991) 16S/23S rRNA Sequencing. In: Stackebrandt, E. and Goodfellow, M., Eds., Nucleic Acid Techniques in Bacterial Systematic, John Wiley and Sons, New York, 115-175.

Leser, T. D., Amenuvor, J. Z., Jensen, T. K., Lindecrona, R. H., Boye, M., and Moller, K. (2002). Culture-Independent Analysis of Gut Bacteria: the Pig Gastrointestinal Tract Microbiota Revisited. *Appl. Environ. Microbiol.* 68, 673–690. doi:10.1128/aem.68.2.673-690.2002.

Weisburg, W. G., Barns, S. M., Pelletier, D. A., and Lane, D. J. (1991). 16S ribosomal DNA amplification for phylogenetic study. *J. Bacteriol*. 173, 697–703. doi:10.1128/jb.173.2.697-703.1991.
